# Zinc oxide nanoparticles using plant *Lawsonia inermis* and their mosquitocidal, antimicrobial, anticancer applications showing moderate side effects

**DOI:** 10.1038/s41598-021-88164-0

**Published:** 2021-04-23

**Authors:** Pandiyan Amuthavalli, Jiang-Shiou Hwang, Hans-Uwe Dahms, Lan Wang, Jagannathan Anitha, Murugan Vasanthakumaran, Arumugam Dhanesh Gandhi, Kadarkarai Murugan, Jayapal Subramaniam, Manickam Paulpandi, Balamurugan Chandramohan, Shivangi Singh

**Affiliations:** 1 Division of Entomology, Department of Zoology, School of Life Sciences, Bharathiar University, Coimbatore, 641 046 India; 2 Institute of Marine Biology, National Taiwan Ocean University, Keelung, 20224 Taiwan; 3 Center of Excellence for Ocean Engineering, National Taiwan Ocean University, Keelung, 20224 Taiwan; 4 Center of Excellence for the Oceans, National Taiwan Ocean University, Keelung, 20224 Taiwan; 5 Department of Biomedical Science and Environmental Biology, Kaohsiung Medical University, Kaohsiung, 80708 Taiwan; 6 School of Life Science, Shanxi University, TaiyuanShanxi Province, 030006 China; 7 Department of Zoology, Kongunadu Arts and Science College, Coimbatore, Tamil Nadu 641029 India; 8 Department of Biotechnology, Thiruvalluvar University, Serkadu, Vellore, Tamil Nadu 632 115 India; 9 Department of Medical Laboratory Science and Biotechnology, Kaohsiung Medical University, Koahsiung, Taiwan

**Keywords:** Biotechnology, Ecology, Environmental sciences, Nanoscience and technology

## Abstract

Microbes or parasites spread vector-borne diseases by mosquitoes without being affected themselves. Insecticides used in vector control produce a substantial problem for human health. This study synthesized zinc oxide nanoparticles (ZnO NPs) using *Lawsonia inermis* L. and were characterized by UV–vis, FT-IR, SEM with EDX, and XRD analysis. Green synthesized ZnO NPs were highly toxic against *Anopheles stephensi*, whose lethal concentrations values ranged from 5.494 ppm (I instar), 6.801 ppm (II instar), 9.336 ppm (III instar), 10.736 ppm (IV instar), and 12.710 ppm (pupae) in contrast to *L. inermis* treatment. The predation efficiency of the teleost fish *Gambusia affinis* and the copepod *Mesocyclops aspericornis* against *A. stephensi* was not affected by exposure at sublethal doses of ZnO NPs. The predatory potency for *G. affinis* was 45 (I) and 25.83% (IV), copepod *M. aspericornis* was 40.66 (I) and 10.8% (IV) while in an ZnO NPs contaminated environment, the predation by the fish *G. affinis* was boosted to 71.33 and 34.25%, and predation of the copepod *M. aspericornis* was 60.35 and 16.75%, respectively. ZnO NPs inhibited the growth of several microbial pathogens including the bacteria (*Escherichia coli* and *Bacillus subtilis*) and the fungi (*Alternaria alternate* and *Aspergillus flavus*), respectively. ZnO NPs decreased the cell viability of Hep-G2 with IC_50_ value of 21.63 µg/mL (R^2^ = 0.942; *P* < *0.001*) while the concentration increased from 1.88 to 30 µg/mL. These outcomes support the use of *L. inermis* mediated ZnO NPs for mosquito control and drug development.

## Introduction

Globally, mosquitoes (Diptera: Culicidae) are threatening human individual and public health as vectors of parasites and pathogens^1^. Mosquitoes provide a substantial threat when compared to other disease-transmitting insects as they spread disease causing pathogens. *Anopheles stephensi* is a vector which transmits the globally most threatful contagious disease malaria^2^. The most serious health problem caused by malaria affects 214 million cases in 2015^3,4^. The appearance of multi-drug resistance of the disease causing protists belonging to *Plasmodium* spp. possess a major obstacle to successful chemoprophylaxis and chemotherapy of this disease^5^.

Then exposure to acoustic vibrations within determined frequency bands leads to dorsal tracheal trunk (DTT) wall rupture in mosquitoes, resulting in the discharge of gases into the body cavity, that block larval development, increase mortality rates or rendering adult mosquitoes flightless. Phyto-constituents that are naturally synthesized by medicinal plants can be utilized for ecofriendly applications in vector control^6,7^. Nanocomplexes using phyto- and microorganisms will minimize the side effects caused by synthetic drugs and also the toxicity to target organisms^8^ in an environmentally friendly manner^9^. Drug resistance provides the main drawback in executing chemotherapy in cancer^10^. The development of efficient versatile drugs against both mosquito-borne diseases and cancer were highlighted^11^.

In spite of increasing evidence for the outstanding mosquitocidal potency of phyto-synthesized nanocompounds and their toxicity against natural predators of mosquitoes, their effects have rarely been studied with respect to sub-lethal doses on predation^12^. Water predators, including juvenile instars of dragon flies, tadpoles, beetles, fishes, and crustaceans^13^. The impact of predatory animals on water bodies is important because such predators were introduced throughout many warm regions of the world for mosquito control^14^. Metabolites with functional groups such as carbonyl, hydroxyl and functional groups of amines, especially the OH-group of flavonoids that react with metal ions lead to a reduced size of metal ions that can be used for nanoparticle synthesis^12^. Zinc oxide nanoparticles can show several morphological varieties such as nanoflowers, nanosheets and even nanorods which were shown to successfully inhibit the growth and development of the bacteria *Escherichia coli*, *Staphylococcus aureus*, and *Klebsiella pneumonia*. Plant derived nanomaterials exhibit various shapes and sizes when compared to nanoparticles produced by other organisms such as algae, fungi, and bacteria^13^. There is no published report that evaluates zincoxide nanocomplex toxicity against the non-target predatory efficiency of *Mesocyclops aspericornis.* Traditional medicine records the plant *Lawsonia inermis* L. as a potential natural dye with numerous medicinal applications. Nanoparticles that are synthesized from the plant materials showed several applications in fields such as medicine, agriculture and the food industry^14^. ZnO NPs (zinc oxide nanoparticles which exhibit remarkable properties such as binding energy (large) and band gap (wide). Wahab et al.^15^ demonstrated applications of multi-dimensional (0D, 1D, 2D) nanostructures for the cytological evaluation of cancer cells and their bacterial responses. These were the properties which made zinc oxide nanoparticles biocompatible, safe, and non-toxic^15^. Zinc oxide nanoparticles are known to have different applications, such as in medical and biological industries, optoelectronics, as antiplatelet agents, anti-inflammatory, anti-angiogenesis, especially as auspicious anti-cancer agents, providing catalytic and semiconductor properties^16^. Zincoxide nanoparticles are known to show insecticidal properties^7^. ZnO NPs were synthesized from *L. inermis* in the present study are also used to resolve the following issues: (a) the lethal effect against the *A. stephensi* malaria vector, to find out the larvicidal and pupicidal effect; (b) the predatory efficiency of the *Gambussia affinis* fishes and small crustacean *Mesocyclops aspericornis* against younger instars of *Anopheles* larvae in ZnO NPs contaminated water environments; (c) antimicrobial potential of nano-formulations against pathogenic microorganism and; (d) *in-vitro* cytotoxicity against the cancer Hep-G2 cells.

## Materials and methods

This study was carried out in compliance with the ARRIVE guidelines (http://www.nc3rs.org.uk/page.asp?id=1357).

Several aspects of below materials and methods are similar to and detailed by our earlier paper Jaganathan et al.^17^. All methods were performed in accordance with the relevant guidelines and regulations of international law and the IAECC of Bharathiar University (see below statement).

### *Anopheles stephensi* cultivation

Details are provided in our earlier paper by Jaganathan et al.^17^ *Anopheles stephensi* eggs were collected from a local breeding habitat, a fresh water tank in Kalveerampalaiyam, Coimbatore (Tamil Nadu, India) and laboratory reared for egg hatching (80% relative humidity and 27 °C and a photoperiod of 14:10 h (L/D). Emerging larvae and pupae were used for toxicological testing as outlined below.

### Leave collection and processing

Plant samples (*Lawsonia inermis*) were collected on a private farm at Maruthamalai hill, Coimbatore, Tamil Nadu, India) with the consent of the farmer. No further legal requirements were necessary for the leave collection of this commercially available plant. The collection of this plant material complied with the relevant institutional (Bharathiar University), national, and international guidelines and legislation. It was authenticated at the Botanical Survey of India, whose voucher specimens number was BSI/SRC/5/23/2019/Tech and deposited at the Department Zoology, BharathiarUniversity. The leaves were rinsed by tap water and dried at room temperature (28 ± 2 °C), and finely powdered. Powdered leave material (10 g) were boiled with 100 mL of double distilled water for further nanocomposite preparation.

### Synthesis of ZnO NPs

The leaf extract was combined with 1-mM ZnNO_3_ (Sigma-Aldrich, India) solution and was stirred at room temperature (35 ± 2 °C) for 1 h. A brown-yellowish precipitate was heated under stirring at 60 °C for 4 h and further, the solution was continuously stirred at room temperature for 24 h^18^. The precipitate was dried at 100 °C. The obtained sample was ground gently using a pestle and mortar and finally, the sample was calcined at 400 °C for 3 h.

### Characterization

The synthesized ZnO NPs samples were analyzed by a UV–vis diffuse reflectance spectroscopy (UV–vis DRS) at a wavelength range of 200–700 nm, using a UV–vis spectrophotometer (Shimadzu—UV 2600, Tokyo, Japan)^19^. Fourier transform infrared spectroscopy (FT-IR) analysis was carried out using a spectrum 65 FT-IR spectrometer (PerkinElmer Co., Ltd., Massachusetts, USA). ZnO NPs were used for scanning electron microscopy (FEI QUANTA-200; SEM), energy-dispersive X-ray spectroscopy (EDX)^18^. XRD pattern were recorded using Cu Kα radiation at a wavelength of 1.54060 Å, with a nickel monochromator in the 2θ range from 10° to 80° using an analytical X-PERT PRO, diffractometer.

### Acute toxicity assessment against *A. stephensi*

In the laboratory the larvae and pupae of *A. stephensi* (I, II, III, or IV instars) were exposed for 24 h at concentrations of 20, 40, 60, 80 and 100 ppm of *L. inermis* broth and 2, 4, 6, 8 and 10 ppm of ZnO NPs in triplicates. Dechlorinated water without acetone served as a control. Using probit analysis (Finney, 1971) LC_50_ and LC_90_ were calculated as follows (Eq. 1):

1 $${\text{Percent mortality }} = \left( {\text{Number of dead individuals/number of treated individuals}} \right) \, \times 100$$

### Biotoxicity assay on *Gambusia affinis*

Details are provided in our earlier paper Jaganathan et al.^17^. Teleost fishes of *Gambusia affinis* were collected from the Tamil Nadu Fisheries Department (Mettur Dam, Salem, Tamil Nadu, India) and maintained at 27 ± 3 °C and R.H. 85% in cement tanks (120 cm diameter, 60 cm depth) filled with field collected water.

### ROS determination

ROS Measurement Intracellular ROS was measured using (DCFH-DA, Himedia). DCFH-DA enters cells dichlorofluorescein, which reacts with ROS to form the fluorescent dichlorofluorescein (DCF). Cells were loaded with 5uMDCFH-DA for 30 min at 37 °C before ZnO NPs treatment. The DCF fluorescence intensity was determined immediately afterwards using spectro-fluorometer with excitation and emission settings of 485 and 530 nm.

### Predation assays under standard laboratory conditions

Details are provided in our earlier paper Jaganathan et al.^17^. Here, predation efficiency of *G. affinis* adults was assessed against *A. stephensi* (I–IV) instar larvae. In each trial mosquitoes, *n* = 200 larvae were introduced with one *G. affinis* adult in a 2-L glass arena filled with dechlorinated water and five replicates were conducted. Control arenas contained dechlorinated water only. All arenas were checked every 24 h for 5 days and the number of prey missing that were assumed to be eaten by mosquito fish was recorded. After each check, the missing mosquito larvae were replaced with new ones. Predation efficiency was calculated by (Eq. 2).

2 $${\text{Predation efficiency}} = \left( {\text{Number of consumed mosquitoes/total number of mosquitoes}} \right) \, \times 100$$

We confirm that the experimental protocol was approved by the here named institutional committee: Institutional Animal Ethical Clearance Certificate (IAEC) of the Bharathiar University, Coimbatore—641046 (see appended original document signed by IAEC Chairman Prof. V. Vijaya Padma).

### Predatory efficiency of* G. affinis* species after treatment with synthesized ZnO NPs

Details are provided in our earlier paper Jaganathan et al.^17^. Predation assays in contaminated aquatic environments: the predation efficiency of *G. affinis* adults was assessed against I-IV instar larvae of *A. stephensi*, after a mosquitocidal treatment with standard and green-synthesized ZnO NPs. For both mosquito species, n = 200 I-IV instar larvae were introduced with one *G. affinis* adult in a 2 L glass tank filled with dechlorinated water plus 1 mL of the desired concentration of NPs (i.e. 5 ppm of ZnO NPs, 1/3 of the LC_50_ calculated against I instar mosquito larvae)^20^. For each mosquito species, three replicates were used. Control was dechlorinated water only. All experimental tanks were checked every 24 h at day and night time and the number of prey eaten by mosquito fishes was recorded. After each checking, the predated mosquito larvae were replaced by new ones. Predation efficiency was calculated using the above-mentioned formula (Eq. 2).

### Predation of* Mesocyclops aspericornis* against malaria mosquitoes

Details are provided in our earlier paper Jaganathan et al.^17^. In this experiment, the predation efficiency of *Mesocyclops aspericornis* adults was assessed against *A. stephensi* larvae. For each instar, *n* = 100 mosquitoes were introduced, with 10 copepods, in a 500-mL glass beaker containing 250 mL of dechlorinated water. Mosquito larvae were replaced daily by new ones. For each mosquito instar, five replicates were conducted. Control was 250 mL of dechlorinated water without copepods. All beakers were checked after 1, 2, 3, 4 and 5 days and the number of prey consumed by copepods was recorded. Predatory efficiency was calculated using the following formula (Eq. 2):

$${\text{Predation efficiency}} = \left( {{\text{Number of consumed mosquitoes}}/{\text{total number of mosquitoes}}} \right) \, \times 100$$

### Predation of* M. aspericornis* against malaria mosquitoe post-treatment with ZnO NPs

Here, we evaluated the predation efficiency of *M. aspericornis* adults against *A. stephensi* larvae, after a mosquitocidal treatment with synthesized ZnO NPs. For each instar, *n* = 100 mosquitoes were introduced with 10 copepods in a 500-mL glass beaker filled with dechlorinated water treated with synthesized ZnO NPs (i.e. for both species, 1/3 of the LC_50_ calculated against first instar larvae). Mosquito larvae were replaced daily with new ones. For each mosquito instar, five replicates were conducted. Consumed by copepods was recorded. Predatory efficiency was calculated using the above-mentioned formula (Eq. 2). Control was dechlorinated water without copepods. All beakers were checked after 1, 2, 3, 4 and 5 days and the number of prey consumed by copepods was recorded.

### Antimicrobial inhibitory assay

All were provided by the Microbial Type Culture Collection and Gene Bank Institute of Microbial Technology Sector 39-A, Chandigarh-160,036 (India). Antimicrobial activity of Li-ZnO NPs was tested against the selected bacteria (*Escherichia coli* and *Bacillus subtilis*) and the fungal strains (*Alternaria alternate* and *Aspergillus flavus*) using the disk diffusion method^21^. The standard inoculum suspension (10^6^ CFU/mL) was streaked over the surface of the media using a sterile cotton swab to ensure confluent growth of the organisms. 10 μL of synthesized ZnO NPs was diluted with two volumes of 5% dimethyl sulfoxide (DMSO), impregnated on filter paper disks that were placed on the surface of the agar plates. Petri plates were kept for incubation at room temperature (27 °C ± 2) for 24 h. Inhibition was measured in millimeters using a photomicroscope (Leica ES2, Leipzig, Germany) and compared with standard positive controls, i.e. tetracycline (for bacteria) and fluconazole (for fungi).

### Cytotoxicity on liver cancer cell lines

Human liver cancer cell line (Hep-G2) was obtained from National Centre for Cell Science (NCCS), Pune and grown in Eagles Minimum Essential Medium containing 10% fetal bovine serum (FBS)^22^. The cell lines were cultured and incubated according to the procedure given and used for further toxicity studies.

#### MTT assay

After 48 h of incubation, 15 μL of MTT (5 mg/ mL) in phosphate buffered saline (PBS) was added to each well and incubated at 37 °C for 4 h. The medium with MTT was then flicked off and the formed formazan crystals were solubilized in 100 μL of DMSO and then measured at 570 nm using a microplate reader^23^. Following the below mentioned formula the cell viability will be calculated (Eq. 3):

3 $${\text{Percentage cell viability }}\left( \% \right) \, = \, ({\text{Mean experimental call absorbance }}\left( {{\text{A}}620} \right)/\left( {{\text{Mean control call absorbance }}\left( {{\text{A}}620} \right)} \right) \, \times \, 100$$

### Data analysis

SPSS 16.0 version was used for all analyses. The average larval and pupal mortality data were subjected to probit analysis for calculating LC_50_, LC_90_, and other statistics at 95% confidence limits, and chi-square values were calculated using the SPSS Statistical software package 13.0 version. Results with *P* < *0.05* were considered as statistically significant.

## Results

**Figure 1:**
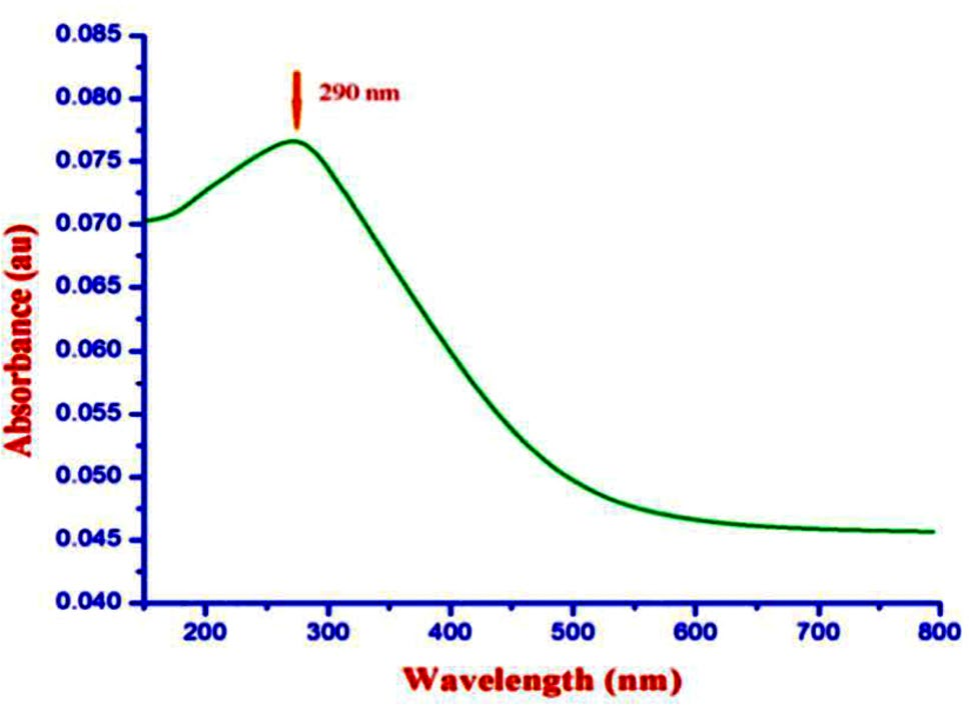
UV–vis- analysis of synthesized ZnO NPs.

**Figure 2:**
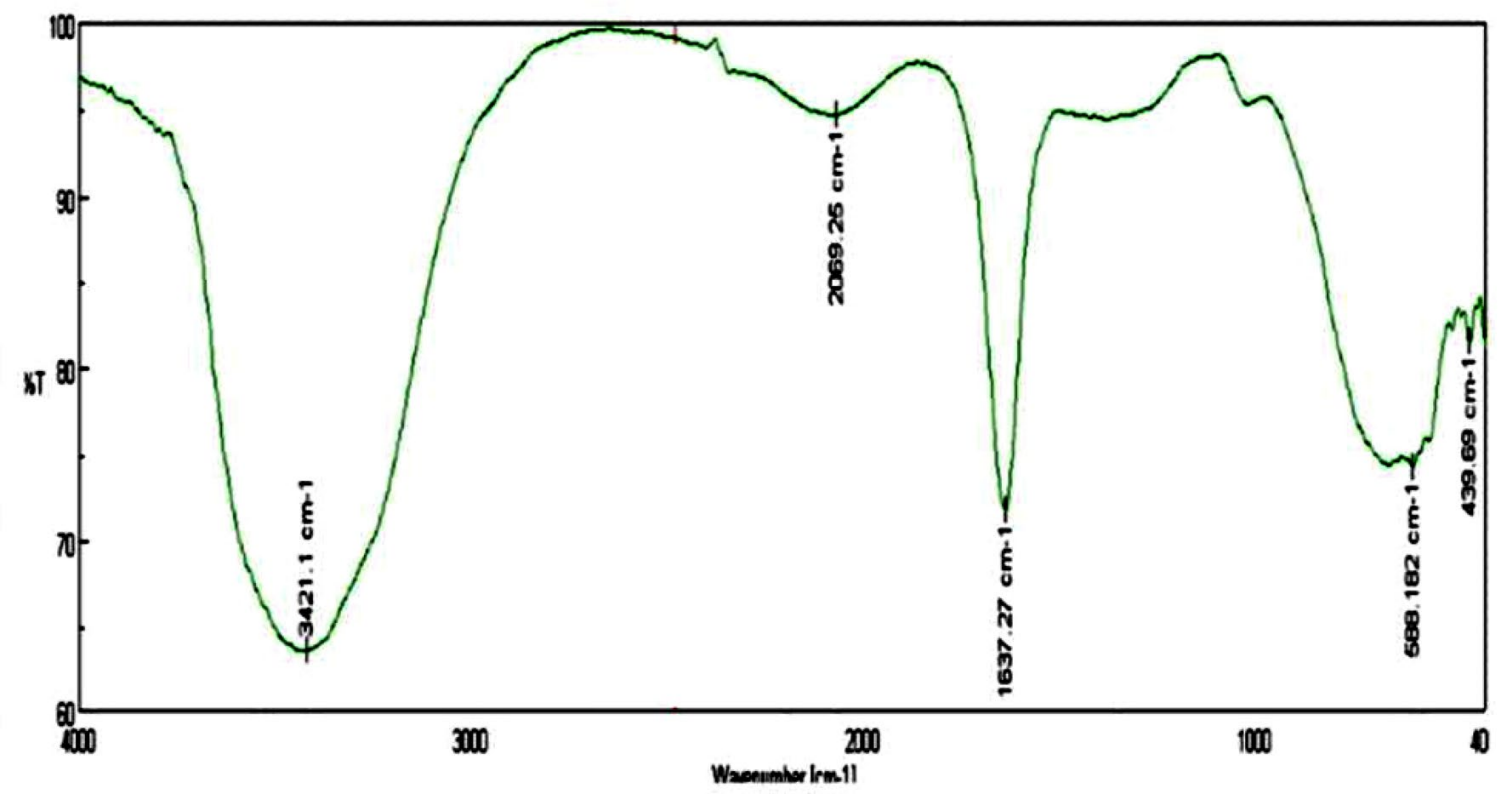
FTIR spectrum of *Lawsonia inermis*-mediated bio-synthesized ZnO NPs.

**Figure 3:**
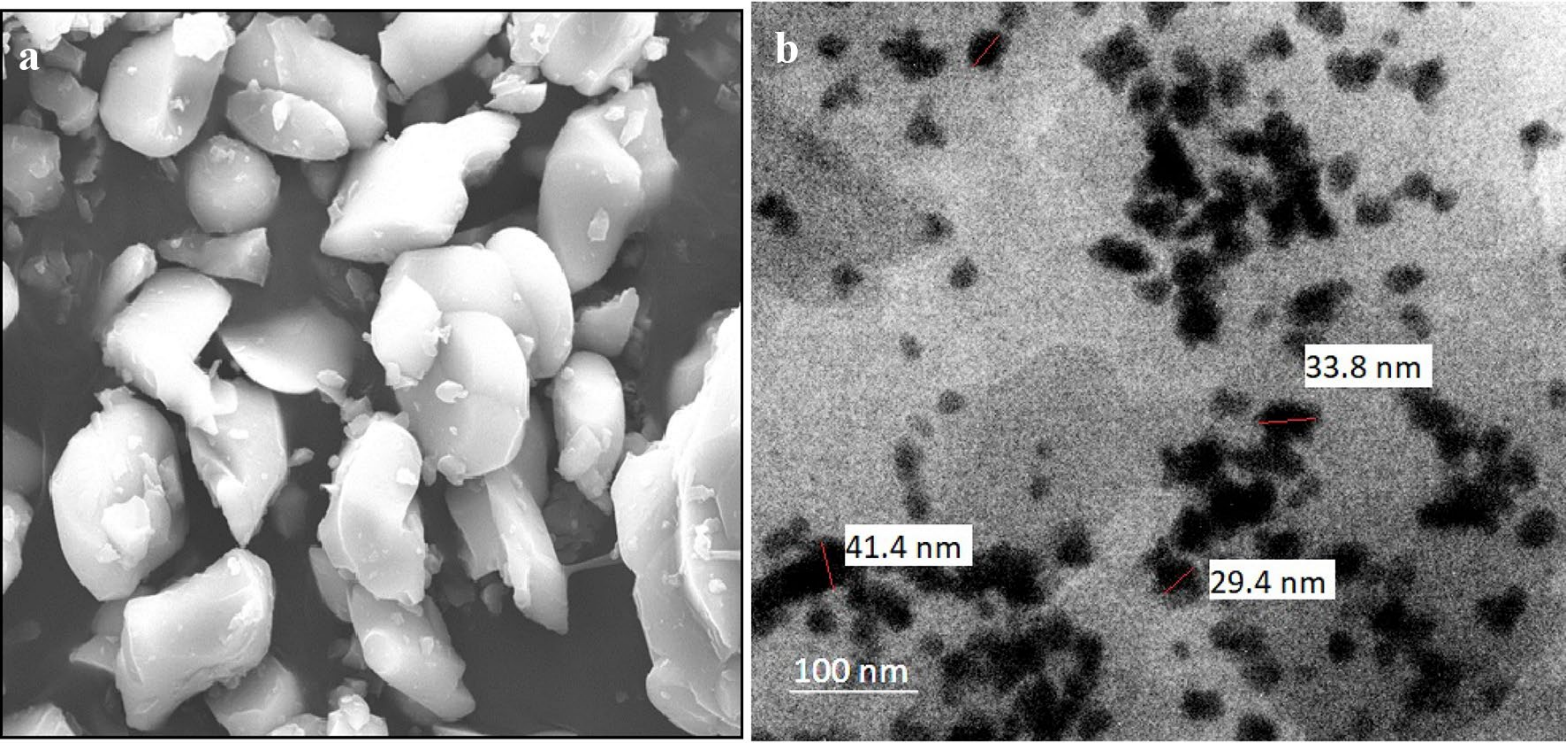
(**a**) SEM images of synthesized ZnO NPs; (**b**) TEM images of synthesized Zno NPs.

**Figure 4:**
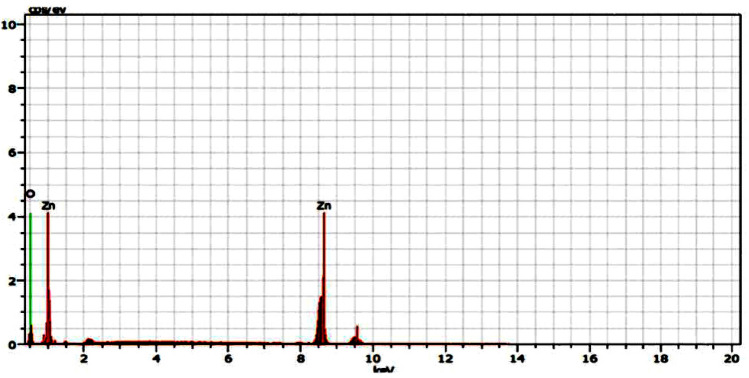
EDX analysis of synthesized ZnO NPs.

**Figure 5:**
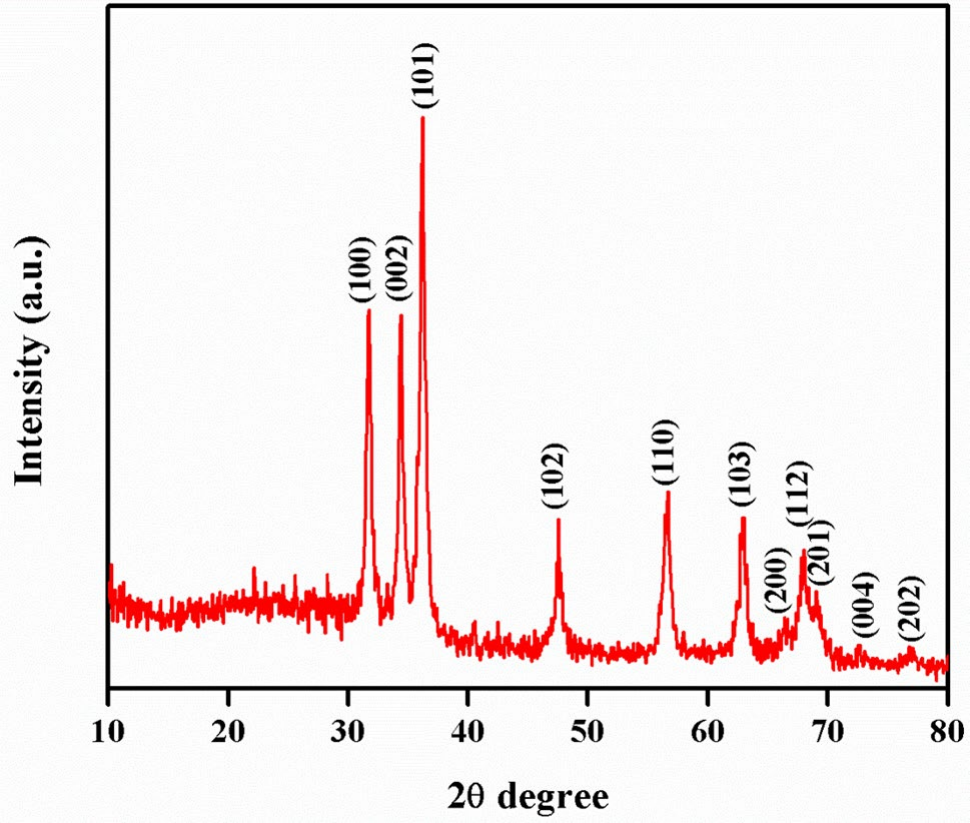
XRD analysis of synthesized ZnO NPs.

As shown in Fig. 1, the absorption spectra obtained for biosynthesized ZnO NP were at 364 nm when investigated under UV-spectroscopy. This absorption was confirmed by the formation ZnO NPs. When *L. inermis* extract was analyzed by FTIR, its spectrum showed several vibration peaks at 3575, 3272, 2073, 1636 and 489 cm^−1^. Thereafter, synthesized ZnO NPs spectrum showed vibration peaks at 1473, 878, 668, and 575 cm^−1^ (Fig. 2). The nanostructure of synthesized ZnO NPs was seen in SEM which acquired 5 nm given in Fig. 3. EDX analysis of synthesized ZnO NPs showed dual peaks which were situated between 1.2 and 8.6 keV, where it is for zinc characteristic lines K and L shell, as shown in Fig. 4. XRD analysis of synthesized zinc oxide nanoparticles showed intense spectra at 2θ = 31.76°, 34.42°, 36.24°, 47.53°, 56.59°, 62.86°, 66.38°, 67.94°, 69.08°, 72.56°, 76.92° parallel to 100, 002, 101, 102,110, 103, 200, 112, 201, 004, and 202 planes, in that order as shown in Fig. 5. Table 1 predicts the mortality of larval instar and pupae of malarial vector (I–IV young ones) when exposed to *L. inermis* at the concentrations of 20, 40, 60, 80, and 100 ppm with the following LC_50_ values: 73.439 (I), 95.204 (II), 110.731 (III), 123.173 (IV), and 131.816 ppm (pupae). Similarly, Table 2 shows the toxicity against larva and pupa of synthesized ZnO NPs. The toxicity was found to be higher at doses of 2, 4, 6, 8 and 10 ppm whose LC_50_ were found to be 5.494 (I), 6.801 (II), 9.336 (III), 10.736 (IV), and 12.710 (V) ppm(pupae).

**Table 1:** Larval and pupal toxicity of *Lawsonia inermis* against malaria vector, *Anopheles stephensi.*

Target	LC_50_ (LC_90_)	95% confidence limit LC_50_ (LC_90_)		Regression equation	*χ*^2^ (*d.f.* = 4)
		Lower	Upper		
Larva I	73.439 (209.586)	61.579 (164.207)	90.861 (319.918)	*y* = 0.691 + 0.009*x*	0.046 n.s
Larva II	95.204 (251.494)	78.996 (188.025)	132.702 (434.667)	*y* = 0.781 + 0.008*x*	0.122 n.s
Larva III	110.731 (249.795)	92.378 (190.816)	153.457(403.497)	*y* = 1.020 + 0.009*x*	0.422 n.s
Larva IV	123.173 (248.584)	102.902 (192.720)	169.426 (385.424)	*y* = 1.259 + 0.010*x*	1.081 n.s
Pupa	131.816 (238.775)	111.249 (189.499)	175.947 (349.963)	*y* = 1.579 + 0.012*x*	0.792 n.s

No mortality was observed in the control.

LC_50_ = lethal concentration that kills 50% of the exposed organisms.

LC_90_ = lethal concentration that kills 90% of the exposed organisms.

*χ*^*2*^ = chi-square value.

*d.f.* = degrees of freedom.

n.s*.* = not significant (α = 0.05).

**Table 2:** Larval and Pupal toxicity effect of ZnO NPs synthesized using *Lawsonia inermis* against the malarial vector *Anopheles stephensi.*

Target	LC_50_ (LC_90_)	95% confidence limit LC_50_ (LC_90_)		Regression equation	*χ*^2^ (*d.f.* = 4)
		Lower	Upper		
Larva I	5.494 (16.661)	4.371 (13.761)	6.487 (22.547)	*y* = 0.630 + 0.115*x*	0.306 n.s
Larva II	6.801 (18.506)	5.774 (15.055)	8.062 (25.809)	*y* = 0.745 + 0.109*x*	0.904 n.s
Larva III	9.336 (23.442)	7.877 (18.025)	12.356 (37.361)	*y* = 0.848 + 0.091*x*	0.192 n.s
Larva IV	10.736 (23.761)	9.070 (18.479)	14.335 (36.609)	*y* = 1.056 + 0.098*x*	0.189 n.s
Pupa	12.710 (25.022)	10.598 (19.416)	17.547 (38.700)	*y* = 1.323 + 0.104*x*	0.905 n.s

No mortality was observed in the control.

LC_50_ = lethal concentration that kills 50% of the exposed organisms.

LC_90_ = lethal concentration that kills 90% of the exposed organisms.

*χ*^*2*^ = chi-square value.

*d.f.* = degrees of freedom.

n.s*.* = not significant (α = 0.05).

Food feeding competence of *G. affinis* fishes were calculated, against I to IV instar larvae of *A. stephensi*. Very small doses of synthesized ZnO NPs were treated with water under standard laboratory conditions where the fishes were introduced, their predation rate subsequent after 24 h was 45% (I) to 25.83% (IV). The food utilization of *G. affinis* was 71.33 (I) to 34.25% (IV), respectively (Table 3). *M. aspericornis* adults predate on *A. stephensi* young larval instars. The predatory efficiency per copepod per day was 4.06, 2.87, 1.79 and 1.08 larvae (I, II, III, and IV, respectively). During post-treatment with sub-lethal doses of synthesized ZnO NPs, the predation efficiency was boosted to 4.06, 2.87, 1.79 and 1.67 larvae (I, II, III, and IV, respectively) (Table 4).

**Table 3:** Predation efficiency of *Gambusia affinis* on *An. stephensi* larvae in standard conditions and post-treatment of ZnO NPs.

Treatment	Prey	Daylight (0–12 h)	Night time (12–24 h)	Total predation (n)	Total predation (%)
Standard conditions	I instar	92.66 ± 2.51	88.33 ± 1.52	180.99	45.00
	II instar	1.66 ± 2.08	72.66 ± 2.08	154.32	38.58
	III instar	76.33 ± 1.52	54.00 ± 2.00	130.33	32.58
	IV instar	59.33 ± 2.51	44.00 ± 2.64	103.33	25.83
Post-treatment with Zn-NPs	I instar	155.00 ± 2.64	130.33 ± 2.51	*285.33*	71.33
	II instar	126.00 ± 2.64	107.33 ± 2.30	233.33	58.33
	III instar	92.66 ± 3.21	79.66 ± 1.52	172.32	43.08
	IV instar	73.00 ± 3.00	64.00 ± 2.00	137.00	34.25

Predation rates are means ± SD of 3 replicates (1 fish vs. 400 mosquitoes per replicate).

Control was clean water without *G. affinis* fishes.

Within the column, means followed by the same letter are not significantly different (weighted generalized linear model*, P* < *0.05*).

**Table 4:** Predation efficiency of *Mesocyclops aspericornis* on *An. stephensi* larvae in standard conditions and post-treatment of ZnO NPs.

Treatment	Targets	Number of consumed preys						Total predation	Consumed preys per copepod per day (n)
		Control	Day 1	Day 2	Day 3	Day 4	Day 5		
Standard conditions	I	0	60.25 ± 1.89	50.00 ± 2.16	38.00 ± 2.16	32.00 ± 2.16	23.00 ± 1.63	203.25	4.06
	II	0	41.50 ± 1.29	36.00 ± 1.15	28.25 ± 0.80	22.25 ± 1.70	15.50 ± 1.29	143.50	2.87
	III	0	26.00 ± 0.81	20.5 ± 0.57	16.50 ± 1.29	14.25 ± 1.70	12.50 ± 0.57	89.75	1.79
	IV	0	20.75 ± 0.50	15.25 ± 1.25	9.75 ± 2.62	5.00 ± 0.81	3.25 ± 0.95	54.00	1.08
Post-treatment with ZnO NPs	I	0	80.25 ± 1.25	66.75 ± 1.70	57.50 ± 0.80	52.00 ± 0.81	45.25 ± 6.25	301.75	6.03
	II	0	53.00 ± 2.16	47.25 ± 1.89	40.5 ± 1.29	32.75 ± 1.70	24.25 ± 1.70	197.75	3.95
	III	0	38.00 ± 1.41	31.75 ± 1.70	28.00 ± 0.81	21.25 ± 1.25	14.5 ± 1.29	133.50	2.67
	IV	0	28.75 ± 1.70	20.00 ± 0.81	14.25 ± 1.25	12.00 ± 0.81	8.75 ± 0.95	83.75	8.37

Predation rates are means ± SD of four replicates (1 *G. affinis* fish vs. 100 mosquitoes per replication).

No predation in control (i.e. clean water without *G. affinis* fish).

Within each column, means followed by the same letter are not significantly different (P < 0.05).

**Figure 6:**
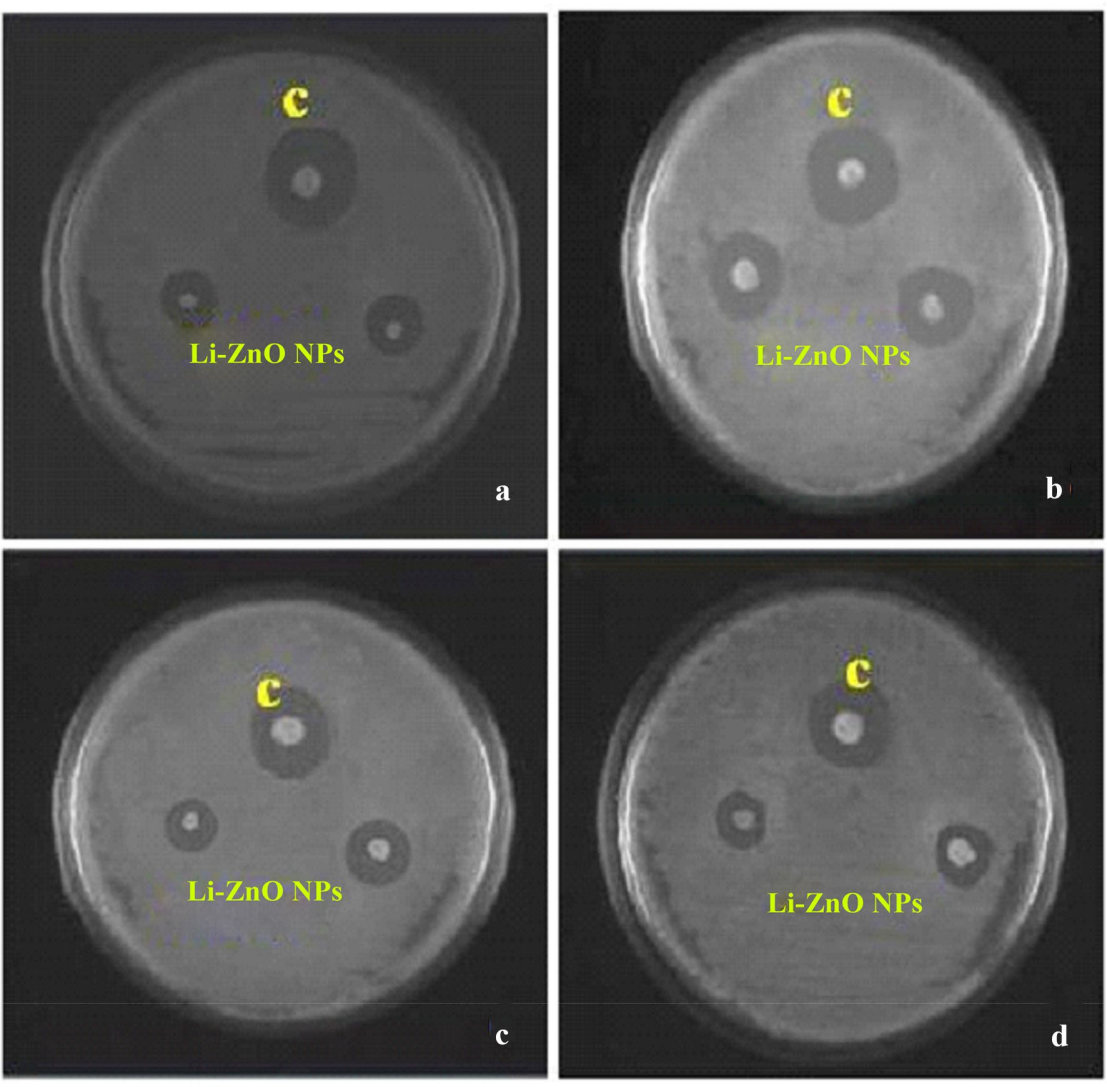
Inhibitory zone of ZnO NPs against pathogenic bacteria and fungi. (**A**) *Escherichia coli*, (**B**) *Bacillus subtilis,* (**C**) *Alternaria alternate*, (**D**) *Aspergillus flavus*; Standard: tetracycline for bacteria and fluconazole for fungi.

**Figure 7:**
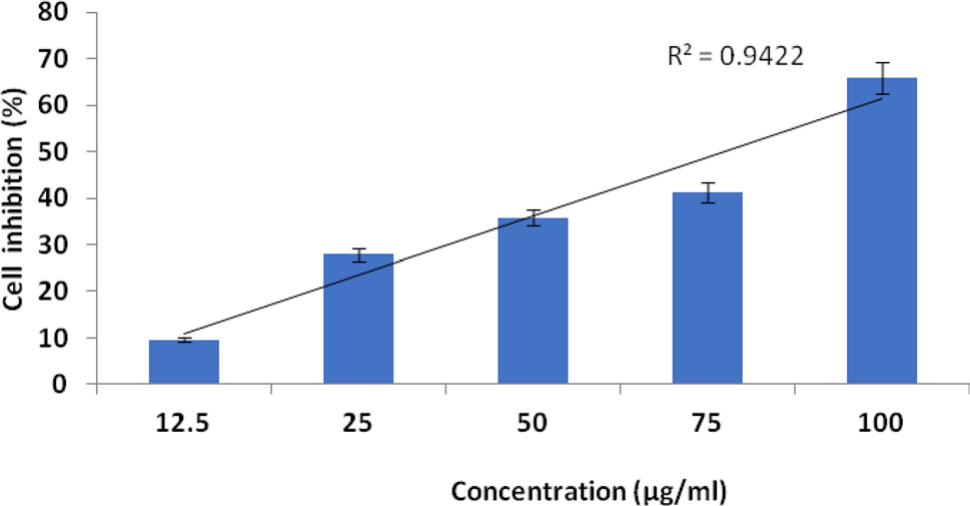
Cytotoxicity of Hep-G2 cancer cell lines mediated by synthesized ZnO NPs: cell growth inhibition (%); above each column, different letters indicate significant differences among treatments (ANOVA, Tukey’s HSD test*, P* < *0.05*).

**Figure 8:**
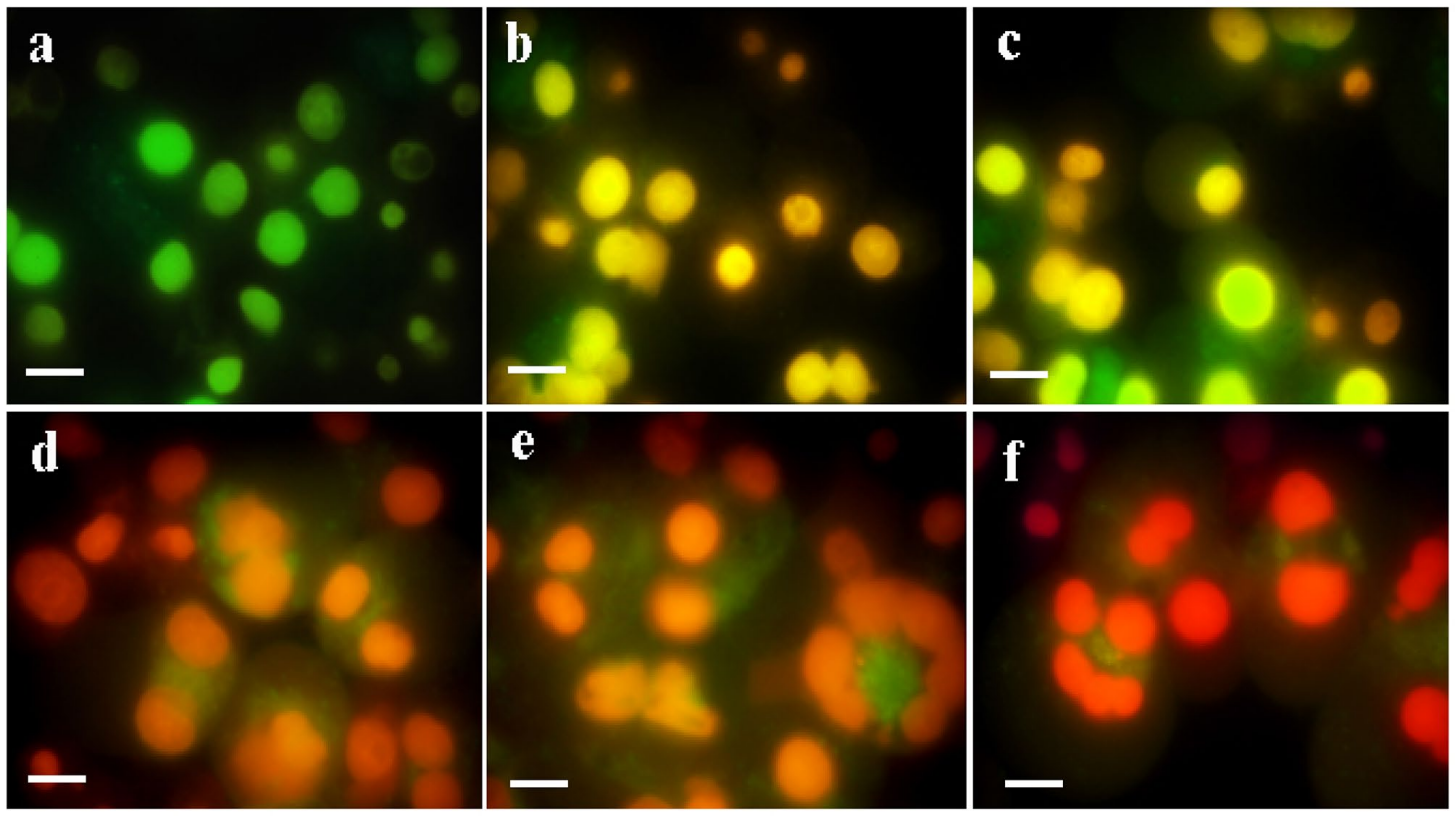
Cell growth inhibition of Hep-G2 cell lines treated with synthesized ZnO NPs. Apoptotic analysis of synthesized nanomaterials on HepG2 cells under fluorescence microscopy (**a**) control; (**b**) 12.5 μg/mL of ZnO NPs; (**c**) 25 μg/mL of ZnO NPs; (**d**) 50 μg/mL of ZnO NPs; (**e**) 75 μg/mL of ZnO NPs; treated cells; (**f**) 75 μg/mL of ZnO NPs; treated cells, the scale bar represents 50 μm.

Antimicrobial effects of synthesized ZnO NPs against selected pathogens like *E. coli* and *B. subtilis* (bacteria) and fungal species like *Alternaria alternate,* and *Aspergillus flavus* were evaluated in the present investigation. Synthesized ZnO NPs were highly effective in inhibiting the growth of *E. coli* (13.3 mm) which were then followed by *B. subtilis* (8.4 mm) respectively given in Table 5 and Fig. 6. Similarly, a maximum zone of inhibition was achieved for the fungus *Alternaria alternate* (11.5 mm), followed by *Aspergillus flavus* (7.8 mm). Synthesized ZnO NPs treated with Hep-G2 cell lines were tested to ensure its cell viability after 24 h. The cytotoxicity on Hep-G2 cell lines mediated by ZnO NPs exhibited a dose-dependent relationship as shown in Fig. 7. In the present study IC_50_ values were found to be 21.63 µg/mL (*R*^2^ = 0.942; *P* < 0.001), respectively, and its morphology and cell inhibition was shown in Fig. 8.

**Table 5:** Zone of inhibition (mm) by *Lawsonia inermis* mediated ZnO NPs against bacteria and fungi.

Target	Zone of inhibition (mm)	
	ZnO NPs (μL)	Standard
Bacteria		Tetracyclin
*E. coli*	13.3 ± 0.5	14.4 ± 0.4
*Bacillus subtilus*	08.4 ± 0.4	10.2 ± 0.2
Fungi		Fucanazole
*Alternaria alternate*	11.5 ± 0.2	13.1 ± 0.2
*Aspergillus flavus*	07.8 ± 0.4	08.1 ± 0.1

**Figure 9:**
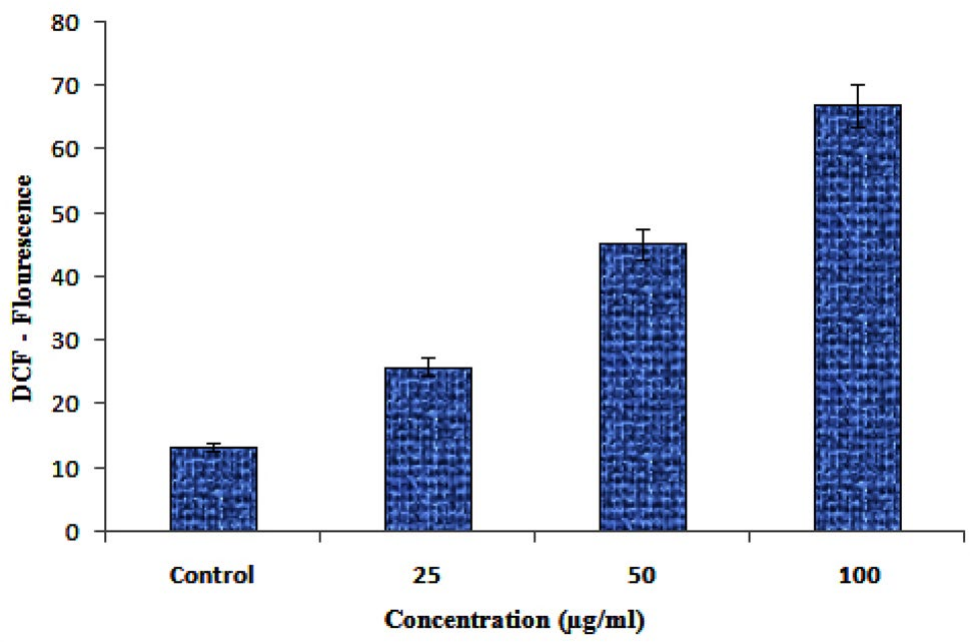
ROS analysis by DCF through ZnO-NPs.

ROS results provided a gradual increase of DCF-Fluorescence from 25 to 100 ug/mL ZnO-NPs concentration (Fig. 9). ROS results provided a gradual increase of DCF-Fluorescence from 25 to 100 ug/mL ZnO-NPs concentration Zinc oxide nanoparticles (ZnO NPs) are well known for their anti-cancer effect due to reactive oxygen species (ROS) generation^57,58^. Therefore, their release into the environment is expected to raise major concern towards ecotoxicity. ZnO nanoparticles have the unique ability to induce oxidative stress in cancer cells, which has been found to be one of the mechanisms of cytotoxicity of ZnO nanoparticles towards cancer cells. This property is due to the semiconductor nature of ZnO. ZnO NPs induces ROS generation, leading to oxidative stress and eventually cell death when the anti-oxidative capacity of the cell is exceeded^59,60^. In the present work apoptotic induction potential of synthesized ZnO NPs were concentrations dependently, when the concentration increases the ROS production also increased and leading to cancer cell death (Fig. 9).

## Discussion

As shown in Fig. 1, a UV–vis DRS analysis of synthesized ZnO NPs showed an absorption peak at 364 nm, revealing a blue shift at a band gap value of 3.40 eV. The direct band gap of the synthesized ZnO NPs was evaluated using Eq. (4);

4 $${\text{Eg}} = 1240/\lambda_{\max }$$

where Eg provides the band gap (eV) and λ_max_ is the wavelength (nm) of the absorption edge within the spectrum. As a red shift indicates an increase in particle size and a blue shift indicates a decrease in particle size. Generally, green synthesized ZnO NPs show an absorption peak at 375 nm and a band gap at 3.30 eV. The higher band gap ZnO NPs was suitable for biological application, which highly promoted the generation of ROS in NPs^17^ (see Fig. 9). In this study, synthesized ZnO NPs exhibited a blue shift of an absorption peak at 364 nm higher ban gap value at 3.31 eV. The same trend was observed with previous reports^24–26^.

FT-IR spectrum of *L. inermis* extract exhibited various stretching and transmittance peaks corresponding to various functional groups including alkyl halides, amine, alkynes, and alcohols. Here, peptide bonds of proteins correspond to N–H and C=O stretching frequencies exhibiting a peak at 3575 cm^-1^. A similar banding pattern at 3402 cm^-1^ was reported by Natarajan et al.^27^ and or alcohols and/or phenols, as well as aliphatic amines support the presence of polyphenols at 1027 ~ 1092 cm^−1^ for C–N vibration^28^. The peaks at 802 cm^−1^, stay for the C–H stretching frequencies within the free catechins in the aromatic ring^29^. The plant extract consists of bioactive molecules which help in the nucleation process and the formation of Zn (OH)_2_. After calcining at 400^0^C, the sample changed from Zn (OH)_2_ into ZnO NPs^23^. The synthesized ZnO NPs spectrum reveal several characteristic peaks at 1473, 878, and 668 & 575 cm^−1^ respectively, being similar in the vibration modes of the functional chemical bonds of C=O symmetric stretching, C–H bending, and weak Zn–O stretching. Evaluating the surface morphology of synthesized ZnO NPs, our results indicate them as spherical structures of an average of 5 nm in size. Typically, the green synthesized ZnO NPs using phytoconstituents and its products attain morphologies like cubic, rod, triangular, spherical, or sometimes they are flat round of sizes ranging between 5 to 35 nm^30^. Different size and shape of ZnO NPs was related to plant species and bioactive compounds^31,32^. EDX spectra indicates O and Zn elements having energy levels of 0.5, 1.2, and 8.6 keV, respectively. Moreover, two additional peaks of Cl and Ag were found as well. The major peak of the sample represented Ag, which was due to the sputter coating process with silver (Ag). XRD analysis of synthesized ZnO NPs demonstrated a well-crystallized structure. The three distinctly high diffraction peaks at 2θ = 31.75°, 34.40°, and 36.25° corresponded to the planes of (100), (002), and (101)^33–35^. This was confirmed by the hexagonal structure of ZnO. This was also matching well with the JCPDS card no. 36-1451.

The toxicity on larval and pupal stages of *A. stephensi* caused by ZnO NPs might be due to the reduction of nanoparticles caused by the plant extract. The tiny NP spheres penetrate cells and interfere with physiological processes such as molting^36^. The present study corroborates with the findings of Gandhi and Madhusudhan^37^*,* who postulated the efficiency of *Momordica charantia* leaves reducing ZnO NPs against *C. quinquefasciatus* and *A. stephensi*. Murugan et al.^38^ found that *Sargassum wightii*-synthesized ZnO NPs were highly effective in killing the larvae and pupae of the malaria vector *A. stephensi*. In contrast, silver nanoparticles were found to be efficient at lower doses against the malarial vector *A. stephensi*—however, only against its larval instars. Equally high toxic effects against larvae and pupae of *A. stephensi.* were provided by leaf extracts from both *S. occidentalis* and *Ocimum basilicum*. A dose dependent effect was in agreement with previous evidences from other plant extracts^39^. Our results clearly indicate that ZnO NPs affect pathogen growth by cell wall disruption. ZnO NPs may reduce surface hydrophobicity of bacterial cells and its oxidative stress-resistance genes were down-regulated, causing finally degradation and the death of cells^40^. ZnO NPs has freshly achieved individual notices concerning possible electronic applications due to its unique optical, electrical, and chemical properties^41^. Its heterogeneous catalytic property might be the cause for bacterial growth inhibition through different mechanisms as known from conventional antibiotics^42,43^. Previous reports of 70–74 postulates higher MIC of ZnO NPs against *E. coli, Listeria monocytogenes, Salmonella typhi,* and *S. aureus* provide supportive evidence for the present microbial evaluation. Most of the fungal strains like *F. solani, A. alternate,* and *A. flavus* had shown antifungal drug resistance^44^. Hence it was in need to formulate nanoparticles with potent chemical and structural nature to overcome such drug resistance^45^.

Similarly, Chobu et al.^46^ demonstrated that *Anopheles gambiae* was less efficiently predated upon by the mosquitofish *G. affinis.* Murugan et al.^47^ noted that the teleost guppy fish, *P. reticulata* actively predates on the larvae of *A. stephensi*. Subramaniam et al.^48^ mentioned another study showing that green synthesized Ag NPs with *Mimusops elengi* did not affect predation rates of the mosquitofish *G. affinis* on the mosquitoes *A. albopictus* and *A. stephensi.* In a paper by Murugan et al.^49^ the predatory efficiency of a single copepod species belonging to *M. aspericornis* was 8.0, 6.3, 0.8, and 0.2 larvae (instar I, II, III, and IV, respectively) per day after a post-treatment with seaweed-synthesized silver nanoparticles. Mahesh Kumar et al.^50^ studied the predatory efficiency of a single adult copepod of *M. thermocyclopoides* being 6.5, 4.6, 0.76, and 0.14 *C. quinquefasciatus* larvae per day (instar I, II, III, and IV, respectively). The predatory efficiency was enhanced to 8.7, 5.9, 1.2, and 0.36 larvae day (instar I, II, III, and IV, respectively) after treatment with *Solanum xanthocarpum* fruit extract. Khooshe-Bast et al.^51^ insecticidal effects of zinc oxide nanoparticles made with *Beauveria bassiana* TS11 on the hemipteran insect *Trialeurodes vaporariorum.*

Several tracemetal based NPs showed effect against different cancer cell lines. Generally, were quantum dot approaches shown as being effective among others in cancer treatment. Ahmad et al. (2020) ^52^ could demonstrate cytotoxicity and cell death induced by engineered nanostructures (quantum dots and nanoparticles) in human cell lines. Gold quantum dots shown to impair the tumorigenic potential of glioma stem-like cells via β-catenin downregulation. Zinc oxide quantum dots were demonstrated as Multifunctional candidates for arresting C2C12 cancer cells and their role towards caspase 3 and 7 genes^53^. ZnO NPs also acted as anticancer agents with minimum dosage as confirmed by the IC_50_ values. In the later study the anti cervical cancer effect of ZnONPs against SiHa cancer cells was evaluated using MTT and apoptosis was evaluated. ZnONPs indicated a suppression of SiHa cells with no cytotoxic effect on normal PBMC cells. Results of cytological staining suggested that ZnONPs induced apoptosis through DNA damage and activation of mitochondria mediated intrinsic pathways. Microwave plasma-assisted silicon nanoparticles were shown to be cytotoxic and to have molecular and numerical responses against cancer cells^54^. Silver nanoparticles and earthworm combinations mediated anti-proliferative activity at increasing concentration as revealed by DNA analysis of Hep-G2 cells^55,56^. In accordance to our present findings were *Amorphophallus paeoniifolius* peels ZnO NP inducing cancer cell apoptosis at higher concentrations^57^. Green synthesized ZnO nanoparticles mediated by *Mentha spicata* extract induce plant systemic resistance against Tobacco mosaic virus (Abdelkhalek et al. 2020)^18^.

Depending on their reactivity and localization, ROS are involved in physiological (termed oxidative eustress) and pathophysiological processes (termed oxidative stress). One major source of increased cellular ROS levels is dysfunctional mitochondria. However, increased ROS levels and, in consequence, an altered redox status of the cell provide a specific vulnerability of cancer cells, which can be used for therapeutic approaches. Recently, two opposing redox status modulating therapies have been tested in clinical trials. In the first approach, antioxidants are used to lower the content of ROS in tumor cells upon treatment of ZnO NPs, subsequently inducing cell cycle arrest and, lastly, apoptosis. The other pro oxidative concept aims to increase the ROS level of cancer cells to an extent that exceeds their survival strategies, and in the end results in an increased apoptotic rate as well^57^. Normally, the imbalance of the pro oxidative and antioxidative systems towards higher ROS levels in cancer cells is still maintained below a cytotoxic threshold. Impairment of the cellular antioxidant system or treatment with exogenous ROS generating agents might exceed a certain ROS threshold, thus resulting in detrimental oxidative stress in the nanomaterial treated cells^58^.

Early apoptotic cells were comparable in abundance to untreated cells up to 12 h after exposure. However, early necrotic cells slightly increased from 12 to 17% at 3 h, although this increase was not significant^59^. On the other hand, viable cells diminished, whereas dead cells significantly accumulated at 12 h. Similarly, ZnO-treated BEAS-2B cells showed an increase in apoptotic cells but not in necrotic cells (Fig. 3C). As these results did not definitively establish apoptosis or necrosis as a driver of ZnO cytotoxicity, we quantified cleaved PARP, an apoptotic marker^60^.

## Conclusion

The emergence of multi-drug resistance of vectors and microbes provides a major obstacle for the management of mosquito vectors, cancer, and microorganisms. Hence, there is an urgent need to develop a novel strategy to combat mosquito vectors, and management of cancer. The biosynthesized ZnONPs tested for the lower concentrations possessed larvicidal and pupicidal antimosquito-activities. Field experiments evidenced in addition that larval and pupal populations were reduced with time (24, 48, 72 h). They also boosted the predatory efficiency of the mosquito-fish *Gambusia affinis* and copepods at the breeding sites. So, it can be concluded that these nanoparticles are most effective in controlling the targeted mosquitoes. Moreover, administration of nanoparticles was shown as one of the vector control measures and further indirectly decreased disease transmission and this way assisted to manage diseases at the community and societal level of larger populations. This kind of bioinsecticide package will further decrease poverty and nuisance in mosquito endemic countries by killing mosquitoes and block disease transmission in a harmonious way and this promotes public health to the society. The same holds for conventional hazards imposed by chemicals in delivery matrices and using nanoparticles to reduce toxicity and side effects of drugs. Moreover, raw materials for the preparation of biosynthesized ZnONPs are easily available, cheap, when compared to other metal oxide nanoparticles like gold, palladium and silver. The final products provided high yields of approximately (85–95%) with high purity (97–99%). Hence, we conclude that the biogenesis of *L. inermis*-mediated ZnONPs could be used as a multipurpose agent against mosquitoe larvae, being antimicrobial and anticancer at the same time for societal benefit in the field of biomedical applications.
